# Correlation of choriocapillaris hemodynamic data from dynamic indocyanine green and optical coherence tomography angiography

**DOI:** 10.1038/s41598-021-95270-6

**Published:** 2021-08-02

**Authors:** Chui Ming Gemmy Cheung, Kelvin Yi Chong Teo, Sai Bo Bo Tun, Joanna Marie Busoy, Veluchamy A. Barathi, Richard F. Spaide

**Affiliations:** 1 Singapore Eye Research Institute, Singapore National Eye Centre, 11 Third Hospital Ave, Singapore, 168751 Singapore; 2 Ophthalmology and Visual Sciences Academic Clinical Program (Eye ACP), Duke-NUS Medical School, Singapore, Singapore; 3 Save Sight Institute, Discipline of Ophthalmology, Sydney Medical School, The University of Sydney, Sydney, NSW Australia; 4 Vitreous, Macula, Retina Consultants, New York, USA

**Keywords:** Blood flow, Retina

## Abstract

To investigate the correlation between posterior pole choroidal blood flow evaluated with digital subtraction indocyanine green angiography and enface optical coherence tomography angiography (OCTA). Imaging in animal study. The anatomy of 2 cynomogulus monkeys was studied. Each monkey was given a 0.75 mg/kg injection of indocyanine green in the saphenous vein. The dynamic angiographic filling sequence was recorded at 15 frames per second using the Heidelberg Spectralis. After image registration, sequential frame subtraction was used to image the dye front moving through the choroid. The OCTA was obtained by frame averaging nine separate choriocapillaris slab flow images obtained from the Zeiss Plex Elite 9000. Posterior pole choriocapillaris filling pattern in relation to the choriocapillaris anatomy as imaged by OCTA. In the posterior pole, the choriocapillaris fills in the pattern of discrete units with variable sizes and shapes. The cycle of dye filling begins in the peripapillary area and progresses toward the periphery in a wavelike manner. This filling pattern repeats in a cyclical manner, consistent with the cardiac cycle. OCTA shows a uniform mesh of vessels. While OCTA shows a uniform meshwork appearance of the choriocapillaris, the dynamic dye angiography suggests an irregular configuration of functional units partitioned by pressure gradients as opposed to structural boundaries. Disturbance of local perfusion pressure within choroidal vasculature may result in abnormal flow patterns, which could be evaluated in the clinic using commercially available equipment.

## Introduction

Nearly every important macular disease somehow involves the choriocapillaris and yet the choriocapillaris is one of the most difficult areas in the eye to assess. The choriocapillaris is a meshwork of densely-packed and interconnected capillaries located below the retinal pigment epithelium (RPE) and provides oxygen and nutrients to the outer retina. The choriocapillaris in the macula region has one of the highest blood flow in the body^1^. Alterations in the choriocapillaris has been implicated in aging and in many retinal diseases^2^. However, the angioarchitecture of the choriocapillaris remains incompletely understood. A multitude of techniques, from animal and human post-mortem studies to in vivo angiography, have previously been employed to provide different perspectives to the understanding of the anatomical and blood flow organisation of the choriocapillaris^3–5^.

Hayreh examined the fluorescein angiographic filling of the choriocapillaris in rhesus monkeys^6^. When slowing blood flow in the eye by increasing the intraocular pressure, choriocapillaris was observed to fill in discrete units showing a polygonal pattern first appearing near the nerve head. The central portion of each polygonal area of vessels filled early followed by the peripheral border. From this, Hayreh surmised the choriocapillaris filled in a lobular pattern and proposed each lobule was surrounded by a venous collecting channel that cumulated the blood from the lobule. This pattern differed from that shown by Hogan et al. on histology, in which there were no borders of collecting veins in the plane of the choriocapillaris^7^. Reports related to the presence of venous collecting channels based on corrosion casts have been inconsistent^5,8^. Flower captured ICGA images using a specially modified fundus camera and prepared subtraction angiography to study choriocapillaris filling and compared with corrosion casts from rhesus monkeys^3^. They concluded that the choriocapillaris appeared as a uniform plexus capillary vessels lying in a plane, whose feeding and draining vessels enter from below^9^. One potential problem with corrosion cast studies is plastic is injected at high pressures through the vortex veins prior to tissue digestion, may affect the appearance of choroidal vessels^10^.

A limitation of previous correlative studies is making a comparison of function while alive to post-mortem post-fixation anatomic structures. Advances in digital indocyanine green angiography (ICGA) have permitted high speed angiography and also digital subtraction of sequential frames as a means of following the dye front of the dye bolus, without the need or potential artefacts caused by increasing the intraocular pressure^11^. Development of frame averaging of optical coherence tomography angiography (OCTA) creates high resolution images of the choriocapillaris, offering the possibility of in vivo comparisons with dye based angiography^12–15^. For precise quantification of choriocapillaris flow voids, it is important to address potential artefacts arising from segmentation, projection and signal loss during image processing. In the current study, we report a set of techniques that can evaluate choriocapillaris blood flow in a way that does not require custom research devices and can be performed safely in vivo. We correlated the in vivo OCTA images of the choriocapillaris with flow dynamics extracted using subtraction of ICGA in monkeys.

## Methods

All procedures were carried out in the SingHealth Experimental Medicine Centre, which is licensed by the Agri-Food and Veterinary Authority (AVA) of Singapore and is fully accredited by the Association for Assessment and Accreditation of Laboratory Animal Care International (AAALAC). The study adhered to the Association for Research in Vision and Ophthalmology (ARVO) Statement for the Use of Animals in Ophthalmic and Vision Research and the National Advisory Committee for Laboratory Animal Research (NACLAR) guidelines in Singapore. Ethical approval was obtained from the SingHealth Institutional Animal Care and Use Committee (IACUC) (Approval Reference Number: 2018/SHS/1443). The study conducted is in accordance with ARRIVE guidelines.

### Animals and protocol

In this study, we used two adult male cynomolgus macaque monkeys (*Macaca fascicularis*), (aged 12–14 years, weight 3–6 kg). All animals underwent a comprehensive ocular examination to exclude prior ocular disease. All examinations and imaging procedures were performed under general anaesthesia with the administration of intramuscular (IM) ketamine hydrochloride (20 mg/kg), IM acepromazine maleate (0.25 mg/kg) and IM atropine sulfate (0.125 mg/kg).

After pupil dilation with 1% tropicamide and anesthesia with topical proxymetacaine (Proparakain-pos 0.5%; Ursapharm), the Tonoshield was used to measure the intra ocular pressure. Indocyanine green angiography was performed with the Spectralis; Heidelberg Engineering, Heidelberg, Germany. IV injection of Indocyanine Green (0.75 mg/kg) via the saphenous vein were performed. Dynamic ICGA was captured in movie mode for the first 30 s after dye injection. Subsequent images were captured at 45 s, and at 1, 5, 10, and 20 min.

### Digital subtraction ICG angiography

The method used in this paper is an updated version of that previously reported, in that instead of isolated still frames^11^, higher speed video frame rates were used. The uncompressed avi movies of the filling were imported into FIJI (available at https://imagej.net/Fiji/Download). The filling sequence was isolated. This image stack was replicated. The last image of one stack was deleted as was the first image of the second stack. These frames were removed because the first possible subtracted image was only possible with frame subtracted from frame 1 and the last possible subtracted image would be the last frame subtracted by the one prior. The stacks then underwent image subtraction. This results in the first image of the original stack being subtracted from the second image and so forth through the stack. The resultant grayscale was remapped to the available histogram space. The images were smoothed with a 3D Gaussian having a radius of 0.7 pixels to reduce noise. Using this technique individual frames can be evaluated at full resolution or the series can be played as a video.

### Frame averaging of choriocapillaris images

OCTA images (3 × 3 mm centered over the fovea) were acquired by trained technicians. We only included images with a signal strength of nine and above. We used the preset segmentation boundaries of the PlexElite system for the superficial (Inner limiting membrane (ILM) to Inner plexiform layer (IPL)) and deep retinal plexuses (IPL to outer plexiform layer (OPL)). (This strategy includes the amalgamated imaging of the intermediate capillary plexus with the deep capillary plexus (DCP)^16^. Use of the term DCP in this article acknowledges this amalgamation.) For segmentation of the choriocapillaris, we selected a slab with boundaries 17 µm below retina pigment epithelium (RPE) to 33 µm below the RPE. This was based on previous imaging of the choriocapillaris in monkeys in which image averaging was used on various depths in the inner choroid^17^. The 17–33 µm slab below the RPE produced the best image quality of the choriocapillaris with the least artefacts. To examine the sclera, we generated further slabs using boundaries of 20–28 µm below RPE and 26–33 µm below RPE. Manual adjustment by placing the segmentation lines at the boundaries as defined above was performed when the automated segmentation was seen to be inaccurate. The superficial vascular plexus (SVP), DCP, and choriocapillaris (CC) slabs were entered into a red–green–blue (RGB) color stack. Once the image sandwiches (z-stacks) are made, as done by a macro^17^, the images are aligned in a three-step process using a FIJI a distribution of the program FIJI with the plugin Register Virtual Stack Slices (https://imagej.net/Register_Virtual_Stack_Slices). This plugin can use several different forms of feature extraction from any given image and then one uses several different methods of registration. The approach used in the current method was one of successive approximation. A rigid model was first used for gross image registration and the non-overlapping portions of the images (caused by variations in fixation) were cropped from the image. Then a non-rigid sequence of least squares registration was done followed by an affine b-spline approach to fine tune the registration^8^.

## Results

**Figure 1:**
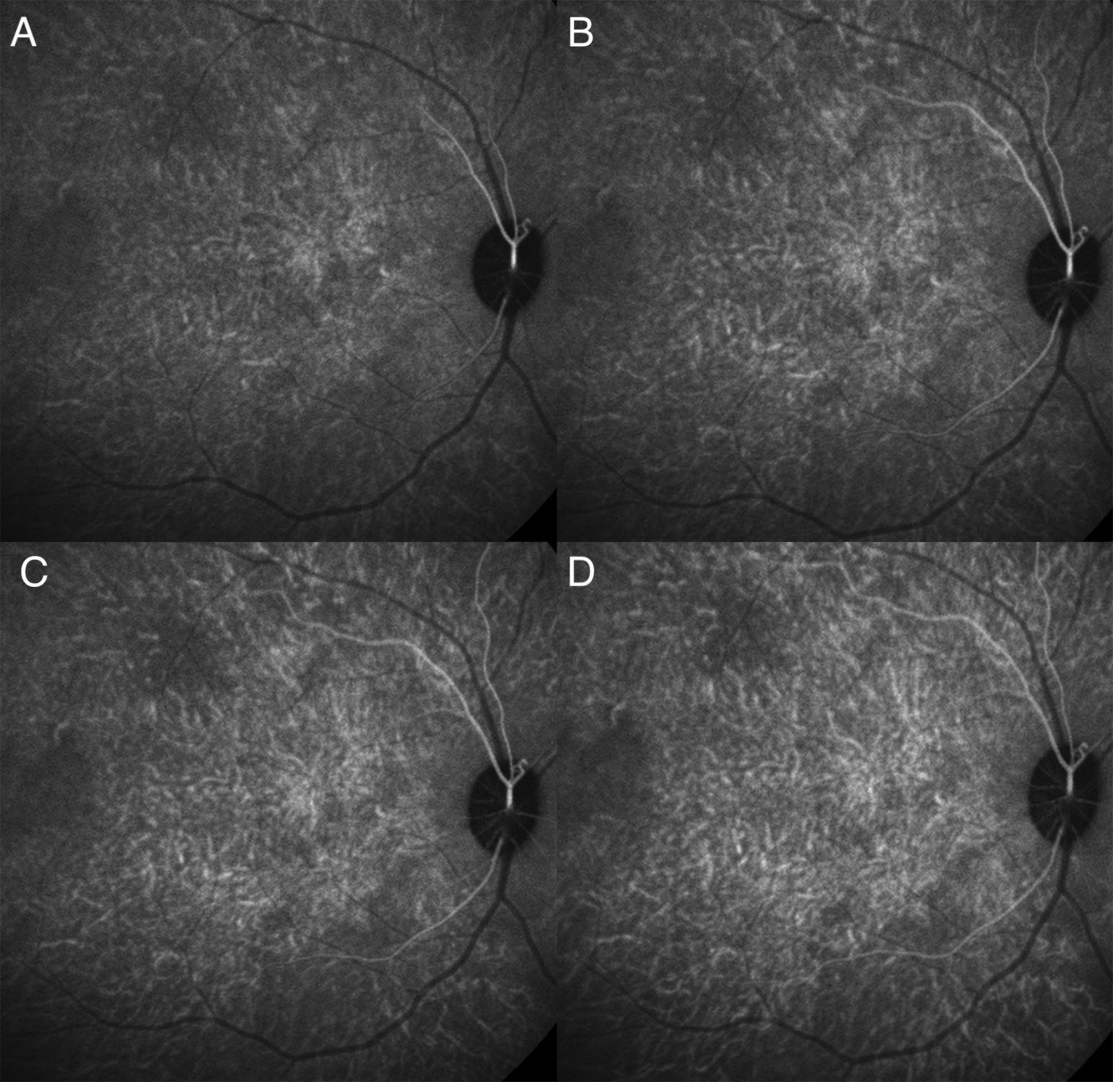
Indocyanine green angiography without subtraction. (**A**–**D**) showed consecutive frames from video angiography. Each frame was 22/100 s apart. Dye first appeared in vessels in the peripapillary and macular area and progressed laterally along individual vessels.

**Figure 2:**
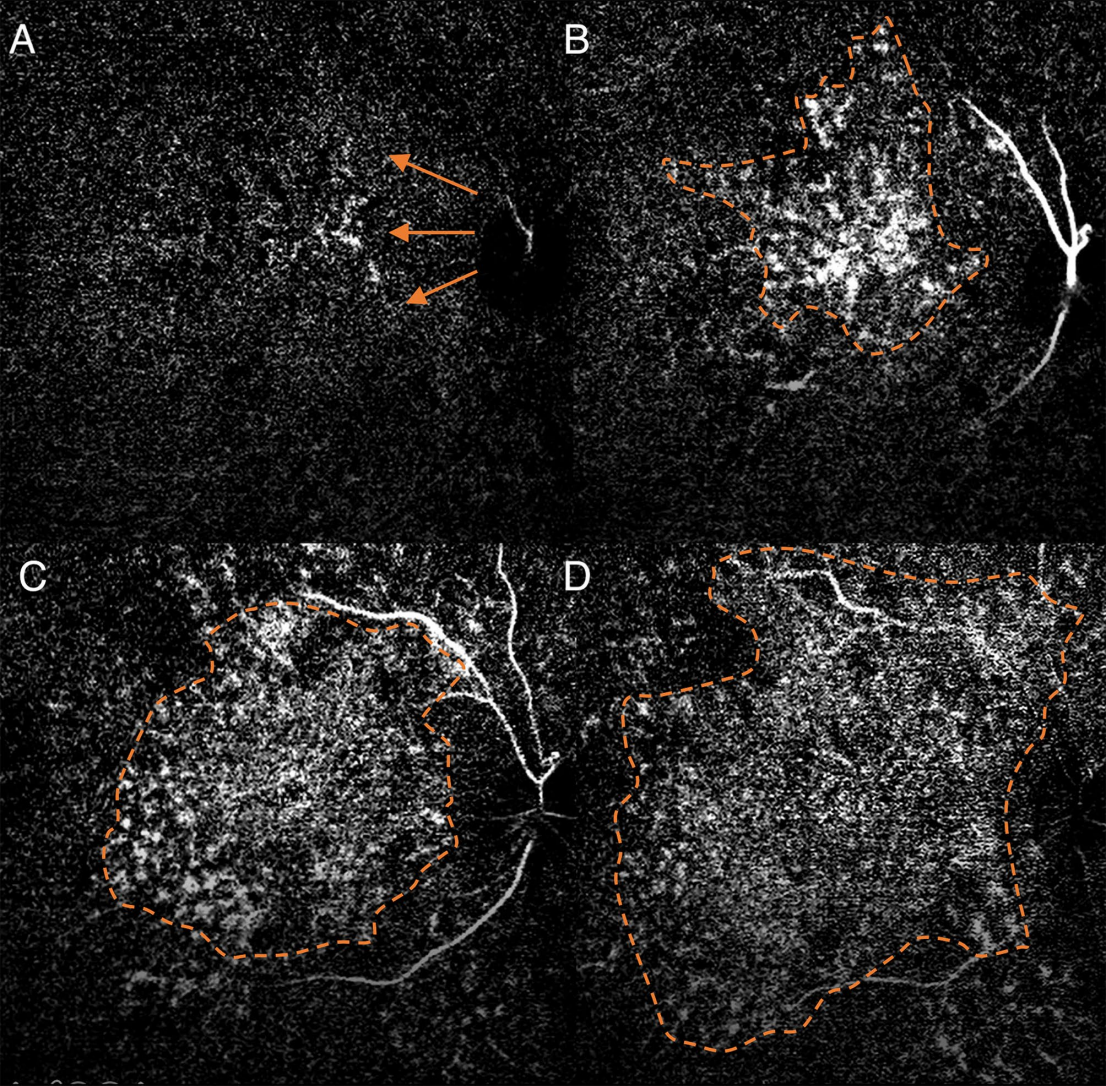
Digital subtraction indocyanine green angiography of the first monkey. (**A**) Dye first entered the peripapillary area of choriocapillaris in regions around the optic disc and posterior pole. (orange arrows). (**B**) A irregular segmented pattern can be seen as the choriocapillaris around the optic disc fills. (**C**) Dye filling progressed radially to the macular region. Many units of variable size and shapes were discernible, separated from each other by dark zone. (**D**) Dye filling in the posterior pole region appeared fairly uniform.

**Figure 3:**
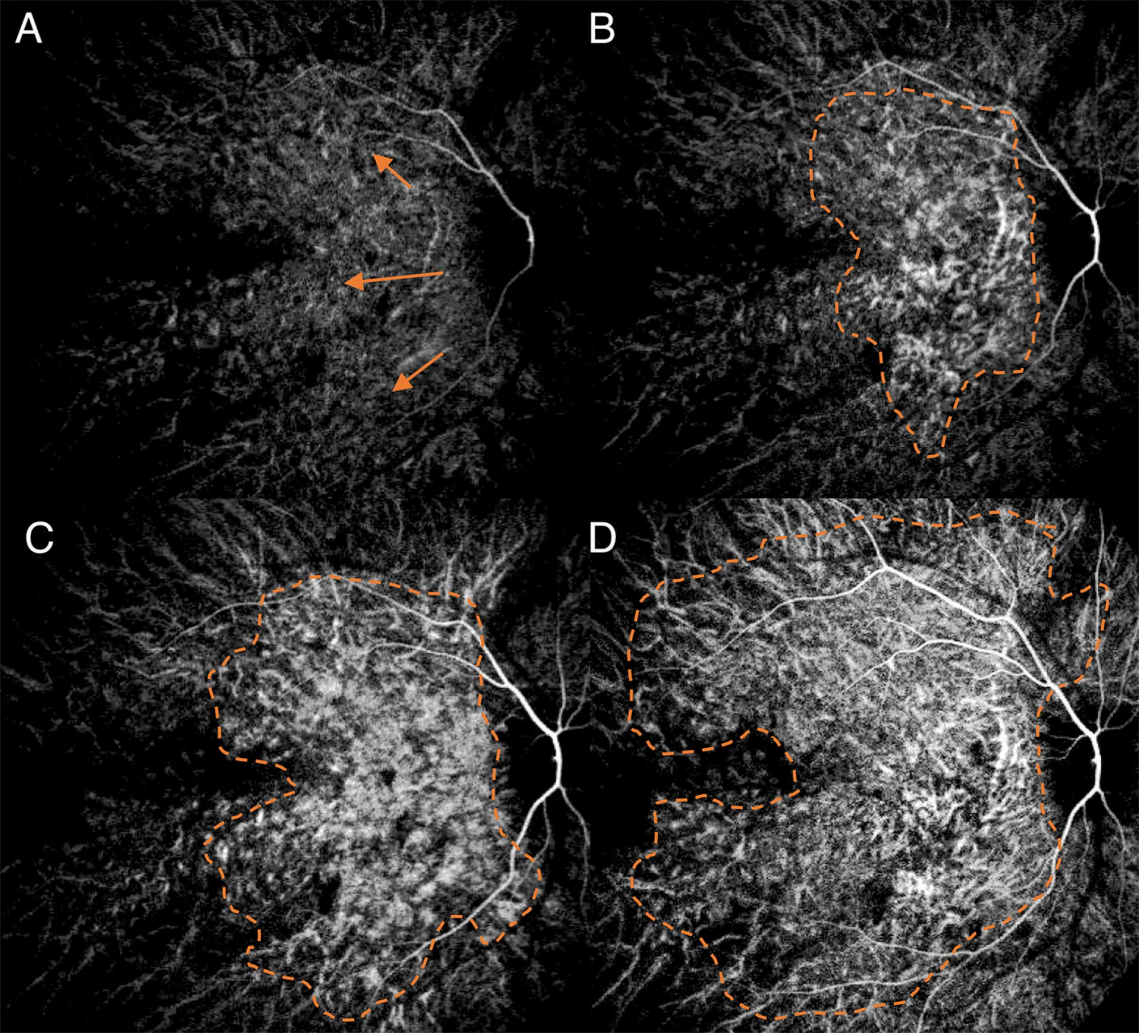
Digital subtraction indocyanine green angiography of the second monkey. (**A**) Dye first entered the peripapillary area of choriocapillaris in regions around the optic disc and posterior pole. (orange arrows). (**B**) Units appear bright around the optic disc. (**C**) Dye filling progressed radially to the macular region. Many units of variable size and shapes were discernible, separated from each other by dark zones. (**D**) Dye filling in the posterior pole region appeared fairly uniform.

**Figure 4:**
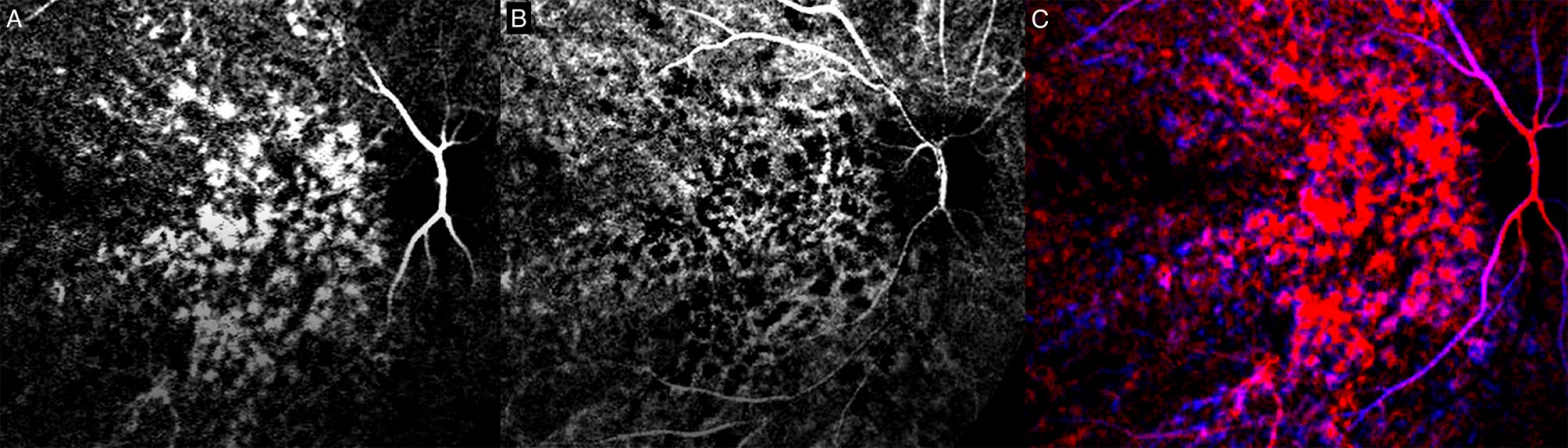
Digital subtraction indocyanine green angiography in which segmented pattern can be seen (same eye as Fig. 2). (**A**) We could define segment (functional lobule) as an area of fast flow surrounded by a perimeter of slow flow. The dye front appears first in the centre of each segmented unit and spread towards the perimeter of each unit in the macular region. In the next frame (0.2 s later), the central area becomes dark (**B**), which implies no change in fluorescence as compared with previous frame. The location of unit correlate well in the two images. The zones between adjacent segmented units appeared faintly brighter in (**B**) compared to in (**A**), which suggests a slow movement of the dye front in this region. Note that the organization of these zones is not regular in relation to the adjacent units. There was also a range of width, estimated between 50 and 200 µm. (**C**) Registration of (**A**—in red) and (**B**—in blue) highlighted the neighbouring units (functional lobules) which can be seen as red areas (fast flow) separated blue areas (slow flow).

We observed a consistent pattern in all the eyes examined. In the non-subtracted ICGA, we observed dye appearance within small choroidal vessels in the peripapillary and macular region immediately after the filling of the central retinal artery. Filling progressed along these vessels from the optic nerve head laterally toward the peripheral retina along the radial path of the vessel from the peripapillary region. The fluorescence also exhibited a pulsatile manner with successive dark times followed by secondary filling (Fig. 1). The subtracted ICGA demonstrated a similar pattern in greater contrast, with the front of dye travelling radially from the peripapillary region to the macular region and then the periphery in consecutive frames. These frames were captured from the first detection of dye over a span of 5–10 s with 0.2 s between frames (Figs. 2, 3). As the first wave of the dye front reaches the peripheral region, the peripapillary region became relatively dark suggesting steady flow in that area. This is followed by a second wave of the dye front entering the peripapillary region in tandem with the pulsatile nature of the blood flow, and the radial progression repeated. Three cycles could be observed, recorded, analysed without any visual changes to the vascular structure or filling suggestive of steady flow. After dye first entered the macular area of choriocapillaris in regions around the optic disc and posterior pole (Figs. 2A, 3A), an irregular segmented pattern can be seen as the choriocapillaris fills in discrete units starting in the peripapillary area and progressed radially to the macular region (Figs. 2B, C, 3B, C). The units of variable sizes were discernible, separated from each other by dark zones that were 50–200 µm wide. In the following frame, dye filling in the posterior pole region appeared fairly uniform, and the peripapillary area appeared dark (Figs. 2D, 3D). The segmented arrangements during different phases of the dye wavefront can be clearly discerned in Fig. 4. Each segment (functional lobule) is seen as an area of fast flow (Fig. 4A) surrounded by a perimetre of slow flow (Fig. 4B) shown as an overlay of red(fast) and blue (slow) flow in Fig. 4C. The dye front appears first in the centre of a quantised area as bright (Fig. 4A). The following subtracted frame shows the centre becoming dark, which implies no change in fluorescence as compared with previous frames, while the zones between adjacent units appear faintly bright (Fig. 4B). This suggests a slow movement of the dye front from centre (red zones) to the perimeter of each unit (blue zones) (Fig. 4C).

**Figure 5:**
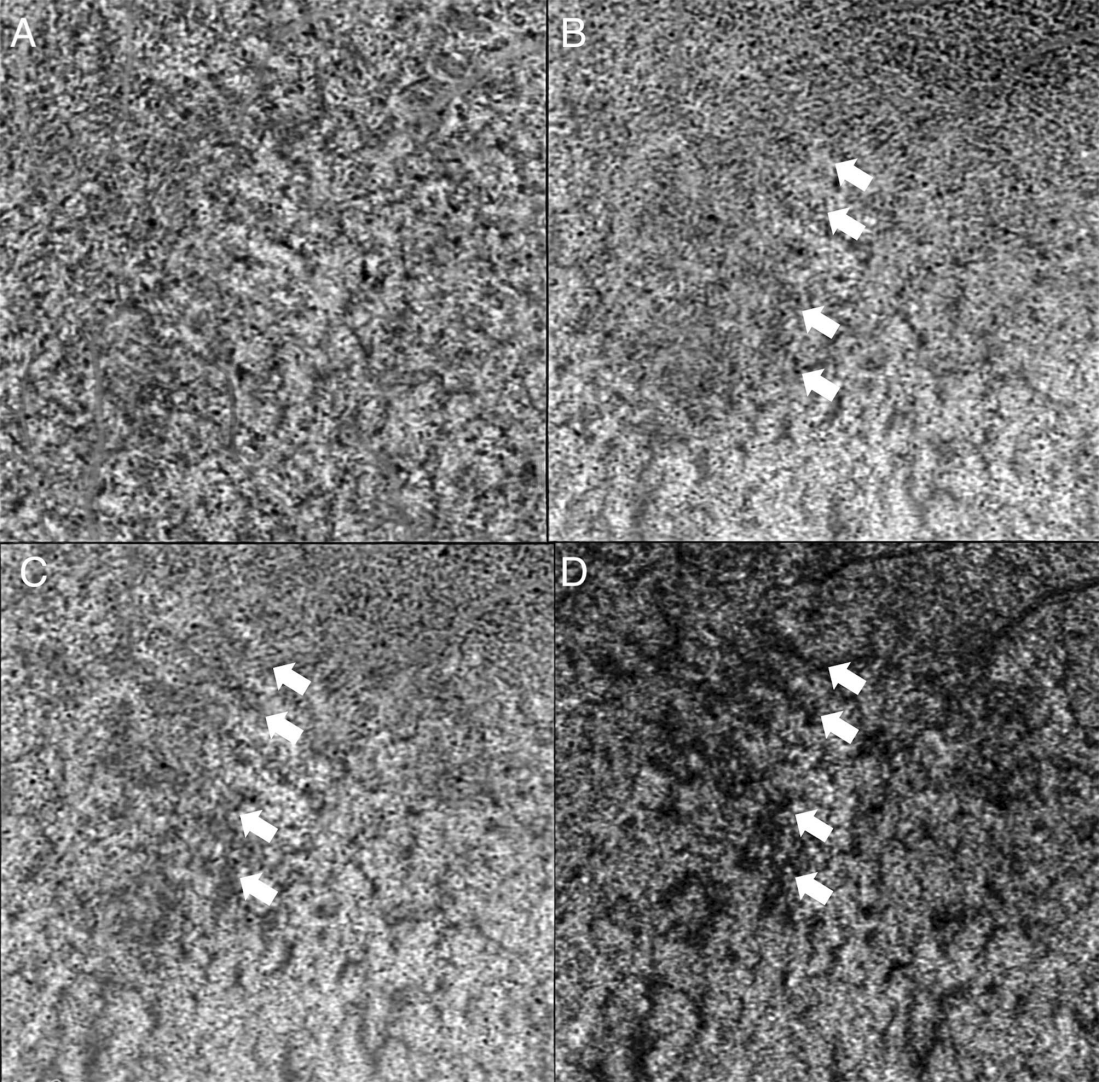
Optical coherence tomography angiography of a 3 × 3 mm area centered over the fovea of the same eye as in Fig. 1. (**A**) showed choriocapillaris slab using boundaries of 17 µm below RPE to 33 µm below RPE. A uniform meshwork of bright areas which appear as continuous densely packed capillary network can be seen. This network appeared to have uniform characteristics in vessel density and size. In particular, a specific lobular or segmented structure was not a dominant feature. Additional slabs to visualize the posterior part of choriocapillaris using boundaries of 20–28 µm below RPE (**B**) and 26–33 µm below RPE (**C**) also showed a uniform meshwork as the dominant feature. Several tubular structures (arrow) can be seen which correlate to choroidal vessels which appear black in the slab with boundaries 25–40 µm below RPE (**D**).

In the corresponding enface OCTA of a 3 × 3 mm area centered over the fovea, a uniform meshwork of bright areas can be seen (Fig. 5A, B). The bright areas appear as continuous densely packed capillary network. This network appeared to have uniform characteristics in vessel density and size. In particular, anatomically distinct lobular structures was not a dominant feature. We did not observe specific physical boundaries delineating independent vascular units, or lobules, when we examined additional thinner 8 µm-thick slabs with a variety of depths of the posterior portion of the choriocapillaris slab (Fig. 5C, D).

## Discussion

In the current study, we used two modalities to evaluate choriocapillaris anatomy and flow characteristics in vivo in cynomolgus monkeys. For the evaluation of choriocapillaris anatomy, we used non-invasive OCTA which has a clear advantage over corrosion casts used in previous studies. OCTA does not involve injection of plastics at high pressure, which may potentially distort the anatomy. In addition, OCTA can be performed and repeated in vivo, thus allowing the possibility to further longitudinal studies of choriocapillaris changes in health and disease in patients. For the evaluation of dynamic filling, the digital subtraction technique we used was applied to images and videos acquired with a Heidelberg Spectralis, a widely available clinical instrument; whereas a specially modified fundus camera, which is not commercially available, was used in previous study by Flower^3^. Both modalities used in the current study are clinically available and can be applied to human patients without the need for any modification.

The dye subtraction technique highlights the difference between one frame and the next in the high-speed video. This method allowed us to evaluate the dynamic changes in blood flow within the first few seconds of choriocapillaris filling. Using OCTA, we examined the flow signal within the choriocapillaris in the macular region with high resolution. We made the following key observations. First, ICGA filling of the choriocapillaris was not uniform. Instead, the dye front first appeared in the peripapillary location and spread radially outward towards the posterior pole and subsequently to the peripheral area. This temporal sequence was observed over several cardiac cycles until blood flow was steady within the choriocapillaris. Second, within the posterior pole, the dye appearance was distributed in individual segmented units with varying shapes and sizes, suggestive of functional units of filling (functional lobules). This observation challenges previously proposed geometry of choroidal lobules with hexagonal outline^6^. These units fill from a central spot and expand radially within a restricted perimeter. Adjacent units are separated by zones which vary in width from 50 to 200 µm in width. The size and brightness within adjacent units was not uniform. Third, OCTA of the choriocapillaris demonstrated a meshwork of densely packed capillaries with no specific anatomical boundaries corresponding to the units observed on ICGA. While these findings are in keeping with previous proposals that the choriocapillaris consists of a freely anastomosing network and the distribution of blood flow within the choriocapillaris is not uniform, our observations of non-uniform size and shapes of each unit and varying width of inter-unit zones led us to additional proposal as follow: In addition to perfusion pressure, we propose novel factors which dictate the dynamics of choriocapillaris flow: (1) the relative distance between arterioles and venules (2) the ratio between adjacent arterioles and venules at each location, (3) the pressure gradient and vector across all the adjacent arterioles and venules at each location. The choriocapillaris is drawn in previous studies as uniform anatomical structure with a central artery and venous collecting channels ringing the perimeter of the anatomic lobule. We found that while the choriocapillaris mesh was uniform, the non-uniform fluorescent patterns can be explained by pressure gradients, which is consistent with recent mapping showing the irregular distribution of feeding arterioles and draining veins in the choriocapillaris.

Our first observation of sequential appearance of the dye front from peripapillary to posterior pole and finally peripheral area can be explained by correlating with the branching pattern of the posterior ciliary artery. Weiter and Ernest examined the vascular supply in 67 human eyes obtained at autopsy through unroofing the orbit or by orbital exenteration and proposed a schema of the vascular supply to the choroid and retina^18^. In most cases, the first branch of the ophthalmic artery was a common trunk that gave rise to both the central retinal artery and the medial posterior ciliary artery. From the medial posterior ciliary artery, short posterior ciliary arteries entered the globe around the nasal side of the optic nerve. Medial to these the medial long posterior ciliary artery then entered the eye. The lateral posterior ciliary artery split into two bundles; the more medial subsequently split into short posterior ciliary arteries that entered into the eye on the temporal portion of the optic nerve and a more lateral branch that split into short posterior ciliary arteries that entered the eye in the macular region and more laterally, the lateral long posterior ciliary artery^19^. Considering the structure of this blood vessels arrangement, a dye front moving through the blood stream would appear first in the area near the optic nerve and then be seen more temporally.

Our second observation of choriocapillaris filling in the macular area as individual units with irregular shapes may be explained by an understanding of the perfusion pressure within the choriocapillaris arterioles and venules. Dollery and coworkers previously described filling of the choriocapillaris in pigs that had their intraocular pressure increased^20^. It was observed that patchy filling began as small dots that expanded slowly, and suggested the choriocapillaris filled as small independent units^20^. Hayreh later used an elevated intraocular pressure model to study the blood flow in monkeys and also proposed a lobular pattern^6^. However, previous reports have not been consistent with the angioarchitecture demonstrated in our study. Hayreh proposed that the boundaries of each lobule was surrounded by specialised venous collecting channels and there was no communication between lobules even though they appeared to be contiguous^6,21–23^. Yoneya and coworkers, who examined plastic injected corrosion casts, concurred with Hayreh’s proposal^24^. These casts were prepared by injecting liquid agents, such as methyl methacrylate, into the vortex veins at high pressure. The material polymerises and the tissue is subsequently dissolved leaving a cast of the vessels. In contrast, other groups have not found venous collecting channels^4,9^. Flower and coworkers found a uniform field of vessels arranged in a mesh without venous collecting channels when pulsed laser excitation ICGA was compared against plastic corrosion cast of the choriocapillaris in rhesus monkeys^9^. These authors hypothesized that choriocapillaris filling patterns were determined by the network of perfusion pressure gradients that exist among the interspersed feeding arterioles and draining venules, but did not comment on their arrangement. They also used methyl methacrylate injected into the vortex veins, potentially distorting the angioarchitecture.

Zouache and coworkers examined the vascular geometry of the choriocapillaris in man and found that the distribution of arterioles and venules was not in an orderly manner as previously thought, but were randomly distributed^25^. In the current study, we examined monkeys in vivo without any disturbances of their ocular physiology. We observed that the filling within each of these units started at the centre and expanded to fill an area within its perimeter. The centrifugal expansion of the dye front within each unit suggests blood is first pushed through an arteriole at the centre of each functional lobule with each pulse cycle. This dye front then expanded through available channels as it follows the pressure gradient between arteries and veins. This is demonstrated in Fig. 4C which explain the sequential movement of dye from the centre to the periphery of a functional lobule following the pressure gradient. Hence in 4b, the bright areas are not single capillary but rather represent areas of slow flow and consequently low pressure gradient. This hypothesis is supported by the observation of varying sizes in these regions which are too large to be vessels and also the criss crossing interconnected patterns that are not in keeping with the vascular architecture of the choroidal venous outflow. Consistent with Zouache’s findings of irregular vascular supply and drainage from the choriocapillaris, we observed that the size and shape of individual units varied in the subtraction ICGA. The size of the intervening zones between filled units, some of which were estimated to be up to several hundred microns in width, were not consistent with the expected size of a vessel. In many of the images from Hayreh’s papers, the spaces between the lobules and the corresponding later fluorescence, both attributed to venous colleting channels, varied greatly in size with some being larger than the retinal arcade veins at the border of the optic disc^6,21–23^. In light of our findings and that of histology of the choriocapillaris^24^, Hayreh’s claims of venous collecting channels appear to be incorrect. On OCTA, we used a range of segmentation slabs with varying thickness and boundaries and did not identify any physical boundaries within the choriocapillaris vascular meshwork.

**Figure 6:**
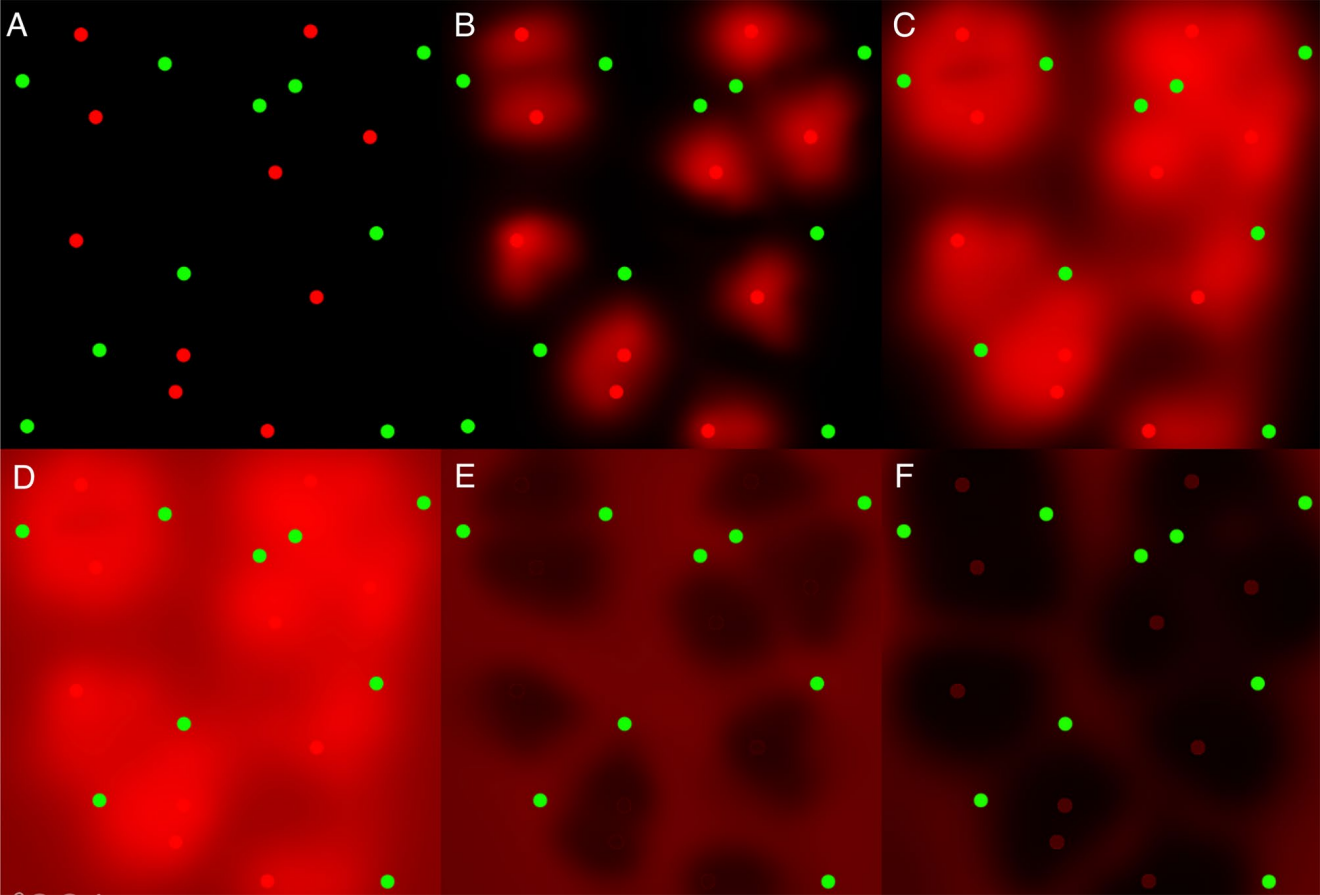
Schematic of the progression in time of blood flow in the choriocapillaris units as observed in this study on ICGA. The red dots represent arterioles and the green dots venules which are randomly distributed (**A**). As blood enters the choriocapillaris via the arterioles, they expand and flow toward venules. The direction and extent of flow is governed by the pressure gradient and distance between the surrounding arterioles and venules (**B**, **C**). The flow from an arteriole outward would be more for a closer vein draining than a distant one. Zones between adjacent units that are last to fill are noted, which also vary in width and shape (**D**). Note that these darker zones do are not centered around draining venules, but represent regions furthest from arterioles. As the blood flow in central part of each unit becomes constant, dye flow was observed in the perimeter of each units and the zones between adjacent units (**E**, **F**).

These new observations are not consistent with previous proposals that suggested the lobular units were the result of arrangement of venous channels which form the boundary around each unit. Our findings further suggest that the arterioles and draining venules are not organised in fixed relation to each other, in terms of number, ratio and arrangement. The resulting perfusion gradient thus is the result of complex interplay between the vector and the pressure differences at any point within the choriocapillaris. As a result, the size and extent of each unit is not physically fixed but is a function of the vector of blood flow. The vector in turn is influenced by the interplay between the ratio and relative spacing between arteries and veins and the pressure within each vessel (Fig. 6).

Understanding of factors which influence choriocapillaris blood flow have important implications. It follows that disturbance of blood flow in one unit can eventually affect blood flow in a neighbouring unit^9,12^. Such flow dynamic may contribute towards the observation that choriocapillaris flow alterations on OCTA follow a power law distribution, showing a progressive reduction in number with increasing size of the flow voids^12,13^. To create a power law distribution, two main factors have to occur: there must be an increase in the number of capillary units affected over time, and the propensity of capillary units to be affected must show some preference for areas around already affected units. Abnormal flow could beget more abnormal flow. This possibility affects more than just aging changes. For example, following focal laser photocoagulation^26^, laser destruction of choriocapillaris in focal areas may affect flow in the immediately adjacent areas as flow cannot occur in the now destroyed capillary units^27^. This may be one of the mechanisms contributing to development of late atrophic scar creep in laser photocoagulation treatment in pathologic myopia or expansion of panretinal photocoagulation scars over time. In eyes with central chorioretiniopathy, choriocapillaris filling defects have long been described on ICGA. Recently, we described intervortex anastomosis in eyes with central serous chorioretinopathy and peripapillary pachychoroid syndrome^27^. Dynamic ICGA study demonstrated low perfusion pressure within these anastomotic segments. Putting together these new observations, we hypothesize that disturbance in perfusion pressure which leads to choroidal congestion may result in choriocapillaris ischemia through the mechanism described in this study^27^.

The strengths of this study include the ability to compare two modalities of imaging the choriocapillaris in vivo. By averaging 9 OCTA, we obtained a high-resolution image of the choriocapillaris. Limitations include the small number of eyes evaluated. Nonetheless, the results were consistent between the two animals. Other factors that could have affected blood flow but not fully discussed here included the blood pressure, endothelial nitric oxide, neural innervation of the pre-capillary arteriole and age of the animals. Only a 3 × 3 mm area was evaluated on OCTA, as the resolution of wider scan areas decrease with current technology. We did not register the OCTA to the ICGA. Our analysis was qualitative and did not include any quantitative parameters. In conclusion, our findings provide new understanding of the dynamic nature of the spatially segmented flow pattern of the choriocapillaris which is governed by pressure gradient. These observations may help to understand observed alterations in the choriocapillaris in various disease states.
